# The appraisal of chronic stress and the development of the metabolic syndrome: a systematic review of prospective cohort studies

**DOI:** 10.1530/EC-14-0031

**Published:** 2014-05-19

**Authors:** N Bergmann, F Gyntelberg, J Faber

**Affiliations:** 1 Endocrine Unit, Department of Medicine O Herlev University Hospital DK-2730, Herlev Denmark; 2 The National Research Centre for the Working Environment Copenhagen Denmark; 3 Faculty of Health Sciences Copenhagen University Copenhagen Denmark

**Keywords:** metabolic syndrome, adiposity, hypertension, dyslipidemia, type 2 diabetes mellitus, psychological stress, job stress

## Abstract

Chronic psychosocial stress has been proposed as a risk factor for the development of the metabolic syndrome (MES). This review gives a systematic overview of prospective cohort studies investigating chronic psychosocial stress as a risk factor for incident MES and the individual elements of MES. Thirty-nine studies were included. An association between chronic psychosocial stress and the development of MES was generally supported. Regarding the four elements of MES: i) weight gain: the prospective studies supported etiological roles for relationship stress, perceived stress, and distress, while the studies on work-related stress (WS) showed conflicting results; ii) dyslipidemi: too few studies on psychosocial stress as a risk factor for dyslipidemia were available to draw a conclusion; however, a trend toward a positive association was present; iii) type 2 diabetes mellitus (DM2): prospective studies supported perceived stress and distress as risk factors for the development of DM2 among men, but not among women, while WS was generally not supported as a risk factor among neither men nor women; iv) hypertension: marital stress and perceived stress might have an influence on blood pressure (BP), while no association was found regarding distress. Evaluating WS the results were equivocal and indicated that different types of WS affected the BP differently between men and women. In conclusion, a longitudinal association between chronic psychosocial stress and the development of MES seems present. However, the number of studies with sufficient quality is limited and the design of the studies is substantially heterogeneous.

## Introduction

The metabolic syndrome (MES) is a cluster of risk factors including male adiposity, dyslipidemia, reduced glucose tolerance and hypertension. MES is highly prevalent and increasing in most parts of the world (1). A meta-analysis has shown that MES increases the risk of myocardial infarction, cardiovascular disease, and cardiovascular disease mortality by twofold (1). Therapies based on lifestyle intervention, especially weight loss, are the main focus in treating MES. However, the etiology seems multifactorial, which calls for new areas of prevention and intervention. One potential risk factor for the development of MES is chronic psychosocial stress, in the following referred to as stress.

In general it is accepted to divide the stress-concept into stressors (strain), stress (the bodily reaction to stressors), and distress (the emotional consequences as reactions to the stressors) (2). Stress occurs when the sum of stressors exceeds an individual threshold. The stress-response is then initiated with activation of the hypothalamic–pituitary–adrenal (HPA) axis and the sympathetic–adrenomedullar (SAM) axis (3). The activation of the two axes takes place in order to maintain the dynamic balance of the body, homeostasis, and is a necessary defence mechanism in situations of acute stress. However, if stress persists a state designated allostatic-load or chronic stress is reached, which among other things results in distress, reduced quality of life, or depression (4). There is no general consensus on how to measure chronic stress. Some have used physiological changes from the activation of the HPA- and SAM-axes as measures of stress: i.e. cortisol, noradrenalin, blood pressure (BP), or resting pulse (5). However, these parameters fluctuate, have great inter-individual variability, and reflect mostly acute stress-load (6). For these reasons, we will not consider the physiological stress-measures as markers of chronic psychosocial stress in this review.

Others have used the number of stressors as a measure of exposure. Example: the number of major life events or hours of work (7). However, the capacity for coping with stressors is dependent on the balance between e.g. the perception of the stressor, individual resources, and coping-mechanisms, and thus the appraised stress caused by the exposure to stressors varies greatly between individuals. This makes the number of stressors by themselves of questionable value as measures of stress. Thus, in the following, we exclude studies measuring only stressors, and focus on studies measuring the appraisal of stress, including reactions to both single and multiple stressors.

The best measure for quantifying the effect of chronic stress is so far by questionnaires (Qs). A variety of Qs have been used for the evaluation of stress. Outside of the working field the most frequently used scale is the Perceived Stress Scale by Cohen. It measures the degree to which situations in one's life are appraised as stressful rated by ten questions on feelings and thoughts during the last month (8). In the occupational area, the most widely used Q's are the Job Demand Control model by Karasek *et al*. (9) and the Effort–Reward Imbalance model by Siegrist *et al*. (10, 11), which both are usable for evaluating work stress (WS). Another way of evaluating stress is by measuring distress which emerges when the allostatic load is reached for the individual subject and only when stress has been in progress for some time. Distress is mostly evaluated by the general health questionnaire (GHQ) by Goldberg & Williams (12), measuring the inability to carry out normal functions and the appearance of distressing experiences.

The aim of the present review is to draw a synthesis from prospective cohort studies, in which chronic stress, measured by Qs, has been evaluated as potential risk factors for the development of MES as well as for the individual components of MES.

## Subjects and methods

### Search strategy

A systematic search in PubMed was done using the following keywords and their combinations: Metabolic syndrome, Abdominal fat, Body mass index, Weight gain, Weight change, Obesity, adiposity, Dyslipidemia, Cholesterol, Blood pressure, Hypertension, Hyperglycemia, Insulin resistance, Diabetes mellitus, Blood glucose, Psychological stress, Psychosocial stress, Work stress, Job stress, Strain, Prospective, Cohort, and Longitudinal.

Web of Science was used to search for references and citations from selected articles.

Articles were limited to human studies from February 1990 to October 2013 written in English. Bibliographies of relevant citations were screened for further articles of relevance.

### Criteria for inclusion

In order to evaluate the causality between chronic stress and the elements of the MES, we included only prospective cohort studies. Studies were included if stress was evaluated by Qs, measuring the appraisal of stress in general (perceived stress) by Q's measuring, e.g. stress from relationships, by appraised WS or by standardized Qs on WS as e.g. effort–reward imbalance or job strain (job demands/control imbalance).

The outcome of the studies should be the diagnosis of MES defined according to general accepted classifications (i.e. from WHO, NCEP, and IDF; Box 1) or by each of the individual components of MES: adiposity, reduced glucose tolerance, dyslipidemia, or elevated blood pressure.

Studies were limited to adults (>18 years of age). When more than two data-categories were reported, the extremes were compared, i.e. ’highest’ vs ’lowest’ category of perceived stress, thus excluding the intermediate groups. When models with different numbers of adjustments were available, the most adjusted model was included excluding the intermediate analyses. When analyses were separated by gender, the individual gender results were included rather than the results based on mixed gender analyses.

### Criteria for exclusion

Studies covering prenatal stress, childhood adverse events, depression, anxiety, hostility, fatigue, or personality types were excluded as were studies measuring the number of stressful life events. Studies measuring physiological stress (such as oxidative stress, cold or heat-shock responses) and studies evaluating stress by a physiological measure such as cortisol or noradrenalin were also excluded. With regard to WS, stress measured as physically challenging work, shift work, or hours of work was excluded to keep the focus on the psychosocial working conditions.

When later follow-up studies were published on the same cohort, any previous reports on the cohort addressing the same subject were excluded unless different exposure or outcome was measured.

The literature review conformed to Preferred Reporting Items for Systematic Reviews and Meta-analysis (PRISMA) statement standards (13). The heterogeneity of study designs and the limited numbers of studies available precluded a meta-analysis.

## Results

Thirty-nine studies were eligible and included. The characteristics and findings of the studies are presented in Tables 1, 2, 3, 4 and 5.

### Association between stress and MES

Seven prospective cohort studies that measured MES as the outcome variable were included: one study evaluated the exposure to global perceived stress (7), two studies evaluated marital stress (14, 15), one study evaluated psychological distress (16), while three studies evaluated the exposure to different measures of WS (17, 18, 19).

The studies not specifically measuring WS were based on two different cohorts: two studies were based on data from The Healthy Women Study Cohort, while one study was based on a cohort of middle-aged white subjects from Finland.

#### Marital dissatisfaction

On data from the Healthy Women Cohort, a study evaluated the 413 women who had answered seven questions on marital quality (14). The study found that after 11.5 years of follow-up, marital dissatisfied women had a three times higher risk of developing MES as defined by NCEP as compared with marital satisfied women (14). Another study based on 216 married couples, living in England, evaluated marital adjustment and found that husbands' report of poorer marital adjustment at baseline were associated with ten times greater risk of wives having MES as defined by NCEP after 4 years of follow-up (15). However, the wives' reports of poorer marital adjustment did not have significant influence on husbands' risk of developing MES (15).

#### Perceived stress

From The Healthy Women Study Cohort, a tendency of a higher risk of developing MES as defined by NCEP and IDF was also found among subjects with perceived stress, evaluated in 523 women after 15 years of follow-up (7). When defining MES by the WHO-5 definitions, the association became statistically significant (7).

#### Distress

From the study based on the Finnish cohort, 466 middle-aged men and women demonstrated that a high score of psychological distress at baseline, evaluated by the General health Q, almost doubled the risk of developing MES as defined by NCEP after a mean of 6.4 years of follow-up (16). The difference remained significant after adjusting for example, socioeconomic status, lifestyle factors, and depression score (16).

#### Work stress

Three studies evaluated WS based on two different cohorts: Two studies were based on the Whitehall II cohort, consisting of working men and women from 20 civil services departments in London. One study was based on the CARDIA study cohort of citizens from Birmingham, Chicago, Minneapolis, and Oakland.

Based on the Whitehall II cohort, one study demonstrated a significantly dose-dependent association between self-perceived stress at work and the risk of developing MES as defined by NCEP (17). The analyses were not separated by gender. The other Whitehall II study investigated feeling of justice at work, and separating the analyses by gender, they found that a high feeling of justice was associated with a lower risk of developing MES during 18-year follow-up among men, but not among women (18). On the CARDIA study cohort, job strain was evaluated among 1408 men and 1558 women. The study showed that after adjusting for sociodemographic factors, health behaviors and depressive symptoms, women in high strain jobs (high demands/low control) had a significantly increased risk of MES compared with women in low strain jobs after 5 years of follow-up (19). The same trend was found among men; however, the association was only borderline significant (19).

#### Conclusion

The studies included support an association between perceived stress and distress and the development of MES. Marital stress was also found to be a risk factor, especially among women. However, only one study measuring stress outside of the working area separated the analyses by gender, and two out of four studies included only women. Concerning WS, perceived stress at work was supported as a risk factor for MES. A high feeling of justice at work was suggested as a protective factor among men, while job strain was found to be a risk factor for MES among women. These findings indicate that the sexes might respond differently to different kinds of stress exposures.

### Association between stress and the single elements of MES

#### Stress and adiposity

Nineteen prospective cohort studies evaluating adiposity were included. One study evaluated the exposure to negative aspects of close relationships, one evaluated strain in relations with family and perceived constraint in life, two studies evaluated self-perceived stress, one stress from daily activities, two evaluated distress and 13 studies on ten different cohorts evaluated WS.

As outcome variables four studies measured weight gain, three measured waist circumference (WC), ten measured BMI, and three measured both WC and BMI.

##### Relationship stress

One study followed over 3000 men and women for 11 years and found that each unit increase in ‘negative aspects of close relationship’ score at baseline slightly increased the risk of a 10% or greater increase of weight gain at follow-up (20). The modest but significant effect was found both when outcome was measured as elevated BMI and as evaluated WC. The analyses were not separated by gender (20). The MIDUS study evaluated strain in relation with family among 1355 men and women and after 9.2 years of follow-up they found family strain to be a risk factor for weight gain among women but not among men (21).

##### Perceived stress

The MIDUS study also evaluated perceived constraint in life and found a positive association between the level of constraints and weight gain among women, but not among men (21). This was supported by the Pitt County Study that found an association between perceived stress and change in BMI among women but not among men after 13 years of follow-up (22). Further a study including only men from one occupation (fire-workers) found no association between self-reported stress (measured by a nonstandardized Q) and weight gain after 7 years of follow-up (23). A study of the Finnish Twin Cohort separated the results both by gender and age, and found in contrast with the other studies that a high level of stress from daily activities was associated with a weight gain of over 10 kg in middle-aged men, but not among men of other age categories and not among women (24).

##### Distress

A study of 466 men and women followed for 6.4 years found high psychological distress to increase the risk of weight gain measured as WC (16). This finding was supported by a study of 1670 men and women, which found psychological distress at baseline to be a predictor for increased BMI after 4 years of follow-up (25). Neither of the studies evaluated the results by gender.

##### Work stress

Three of the 13 studies on WS did not separate their analyses by gender (26, 27, 28). Two of these studies did not find a significant association between WS (effort–reward imbalance and job–demand control imbalance respectively) and increase in BMI (26, 27), while one study found effort–reward imbalance but not job strain to be significantly associated with change in BMI (28).

From the ten studies separating the analyses by gender, five did not support an association between WS and change in weight (measured as weight change or change in BMI) among either gender (29, 30, 31, 32, 33), whereas two studies supported an association between BMI and WS among both genders (21, 34).

Concerning other measures of WS, a study evaluated the feeling of justice at work and found that a feeling of high justice was associated with a lower risk of a large WC (>120 cm for men and >88 cm for women) after 18 years of follow-up among both men and women (18). A study on 3982 female and 152 male Danish health care workers evaluated other psychosocial work characteristics association with change in BMI and among other results they found role conflicts to be associated with a small increased risk of weight gain among women but not men (35).

##### Separating results by age

Two studies separated their results by age and found age-dependent associations: one study found a high level of stress to be a predictor of major weight gain among middle-aged men (30–54 years old), but not among women and among younger age categories of any gender (24). Likewise, in a 5-year cohort of 4424 men and 5488 women, job strain was found to be significantly associated with change in BMI among middle-aged women, but not among women of other age categories and not among men (36).

##### Separating results by baseline BMI

In a re-analysis of the Whitehall II cohort, subjects were stratified according to BMI at baseline (32). The study found that men with BMI <22 at baseline experienced a weight loss during WS, whereas men with a BMI >27 increase in weight (*P*<0.006) (32).

##### BMI vs WC

Three studies included both BMI and WC as outcome measures, and found that the association between stress and adiposity might be dependent on the outcome measure chosen: one study found job strain to be associated with increased BMI among both men and women, but found job strain to be associated with WC only among men (34). Another study found high job strain to be a risk factor for increased WC but not for BMI among both genders (31). While a study on the Whitehall II cohort, not separating the results by gender, found negative aspects of close relationships to be associated with both increase in WC and in BMI after 11 years (20).

##### Conclusion

Studies regarding stress from relationships and overall perceived stress generally supported an association between stress and weight gain, especially among women. Distress seems supported as a risk factor for weight gain among both genders. Concerning the association between WS and weight gain, the studies found conflicting results, with approximately half of the studies finding no association, and the other half finding some association among either men or women. Differences have been found between studies measuring WC or BMI as outcome variables. One study found WS to have a bidirectional effect causing both weight gain and weight loss depending on baseline BMI, which might also contribute to the conflicting results found.

#### Stress and dyslipidemia

We included four studies (on four different cohorts) which prospectively examined the influence of chronic stress on blood lipids: one study focused on perceived stress, one on distress, and two studies focused on WS.

##### Perceived stress

A study of 7066 men and women followed for 10 years found no association between perceived level of stress and lipid levels at neither baseline, nor at follow-up (37).

##### Distress

One study found high psychological distress to be a risk factor for low HDL cholesterol levels after 6.4 years among 466 men and women (16). A tendency of a higher risk of elevated triglycerides was also found, although this was not statistically significant (16).

##### Work stress

Concerning WS, one study followed 4398 men and 1923 women for 18 years and found that low justice at work was associated with the development of reduced HDL cholesterol and elevated triglycerides among men but not among women (18). Another study evaluated job strain and effort–reward imbalance among 545 men and 267 women and found high baseline job strain to be associated with a higher total cholesterol level after 25 years while no association was found regarding effort–reward imbalance (28).

##### Conclusion

Three out of four studies available supported some association between stress and the development of dyslipidemia. These limited data call for further studies in order to obtain a firm conclusion.

#### Stress and type 2 diabetes mellitus

Sixteen prospective cohort studies, with type 2 diabetes mellitus (DM2) as outcome variable, were included. Five studies evaluated the exposure of perceived stress, three evaluated distress, while eight studies focused on WS. The studies focusing on other parameters than WS were all based on different cohorts. Three of the included studies on WS were based on data from the Whitehall II cohort, while the remaining five studies were based on different cohorts.

##### Perceived stress

In a study of 7066 participants men but not women, with a high level of general stress had more than two-times increased risk of developing DM2 during 10 years follow-up (37). This was supported by a study of 55 826 men and women, which found that men, but not women, with a high level of perceived mental stress had a 1.4 higher odds of developing DM2 during 10 years of follow-up as compared with men with a low stress level (38). A higher risk of incident DM2 after 3 years of follow-up was also found among male Japanese employees rating a high level of stress in daily life as compared with employees rating no stress in daily life (39). Likewise, a study of 7251 men showed that self-perceived permanent stress (during the last 5 years) increased the risk of incident DM2, with a hazard ratio of 1.4 after 35 years of follow-up (40). However one study on 3759 Australian men and women did not find an association between high level of perceived stress and development of abnormal glucose tolerance (measured by oral glucose tolerance test (OGTT)) among men, but did find an association among women (41).

##### Distress

One study included 2127 men and 3100 women and found that, after 10 years, men, but not women, with a high level of distress had an increased risk of developing DM2, as compared with persons with low level of distress (42). Another study measuring distress among 9514 men and women found an association between psychological distress and self-reported incident DM after 18 years of follow-up not separating the results by gender (43). However, the association became nonsignificant after controlling for socioeconomic status, e.g. educational level and income, and health status (43). A study on 466 men and women evaluating distress did not support an association between distress level and risk of having fasting glucose ≥5.6 mM after 6.5 years of follow-up (16).

##### Work stress

A study on middle-aged Swedish men and women found job strain to be associated with a decreased risk of DM2 among 2227 men after 8–10 years of follow-up, while no significant association was found among the 3205 included women (44), although this has been disputed recently by Brunner & Kivimaki (45). Nevertheless, this suggested protective effect of job strain found among men has also been found in a subgroup analysis of the Whitehall II cohort (46): when stratifying the results by baseline BMI, WS was found to be associated with a lower risk of developing DM2 among men with BMI <30, but a higher risk of DM2 among women with BMI >30 (46). No association was found between WS and DM2 among obese men and nonobese women and no association was found when the cohort was not stratified by BMI (46). Another study on 2597 Japanese male company employees found no association in either direction between job strain and the risk of developing DM2 after 8 years of follow-up (47).

With regard to control–demands imbalance the above-mentioned study on middle-aged Swedish men and women also separated WS into high demands/low decision latitude (44). In the study, they found no association between high demands and low decision latitude and DM2 among men, while in women it was associated with a four times increased risk of DM2 (44). In contrast, the Nurses Health Study found no significant association between high demands/low decision latitude and incident DM2 after 5.7 years of follow-up on 62 574 women (48). Regarding job control, another study found a significant association between low job control and development of DM2 among women but not among men after 9 years of follow-up (49). The same study found no association between job demands and incident DM2 among either gender (49). Likewise, a study on 8630 men and women from the Whitehall II cohort found no association between high job demands and DM2 among neither men nor women after 10 years (50). A study of 5843 men and 1449 women found no significant association between job control and DM2, however, not stratifying their results by gender (51).

From the Whitehall II study cohort, data on justice at work and effort–reward imbalance were also evaluated: no association was found between level of justice and incident DM2 after 18 years of follow-up (18). In contrast effort–reward imbalance was found to be a risk factor among men but not among women after 10 years of follow-up (50).

##### Conclusion

Perceived stress and distress seemed to be risk factors for the development of DM2 among men but not women. The studies on WS showed conflicting results. Overall WS seemed not to be supported as a risk factor for DM2 among either gender, nevertheless two studies supported, respectively, high demands/low decision latitude and job strain as risk factors among women.

#### Stress and hypertension

Fifteen studies on chronic stress and hypertension have been included. Two studies evaluated the exposure to marital stress on two different cohorts. On individual cohort one study evaluated perceived stress in general and one evaluated distress while 12 studies evaluated exposure to WS based on ten different cohorts. One study evaluated both marital stress and job strain in the same cohort (52).

Six studies evaluated BP by use of ambulatory monitoring (i.e. continuous BP monitoring), seven studies used office BP and two studies measured incident hypertension defined by start of using antihypertensive medication during follow-up. Ambulatory BP has been found to better predict clinical cardiovascular outcomes than office BP and is regarded as the most reliable and reproducible method (53).

##### Marital stress

One study found that among 103 men and women, low marital cohesion and/or satisfaction was associated with a significantly but small increase in 3-year 24-h diastolic and systolic ambulatory BP (DBP and SBP) (54). The other study evaluated the effect of double exposure of both marital cohesion and job strain (52). The study found that subjects with both high job strain and low cohesive marriage had a small but significant increase in ambulatory SBP during one year, while those with high job strain and a highly cohesive marriage had a small but significant reduction in SBP during 1 year (52). However, in subjects without job strain, BP decreased during the year both among the subjects with and without cohesive marriages (52).

##### Perceived stress

A study on 7066 men and women found that participants rating the highest level of perceived stress at baseline had a small but significantly increased risk of starting on anti-hypertensive treatment during the 10 years of follow-up (37).

##### Distress

A study on 466 men and women did not find an association between distress and incident hypertension defined as an office BP ≥130/85 after 6 years of follow-up (16).

##### Work stress

Seven studies evaluated job strain of which five did not divide their results by gender: one study included 11 777 men and 49 145 women and found no significant association between job strain and chronic hypertension, after 3.5 years of follow-up, when hypertension was based on health insurance examinations (office BP ≥190/95) (55). This null association was also found in a study on 209 men and women followed for 5 years, evaluating job strain as a risk factor for increased ambulatory SBP or DBP (56). Likewise, a study based on the Whitehall II cohort found no association between job strain group and change in ambulatory SBP or DBP, among 5630 men and 2456 women followed for 11 years (57). The study on double exposure from marital cohesion and job strain, however, found a significant association between job strain and change in 24-h ambulatory BP after 1 year of follow-up (52).

Two studies on job strain divided their results by gender and both found job strain to be associated with increased office BP among men but not among women (58, 59). In line with these results, a study including only men found those participants that experienced high job strain at baseline but had no job strain at follow-up to have a significant decrease in mean ambulatory BP at work and at home (60). However, those with no job strain at baseline but with job strain at the 3-years follow-up and those with the same job strain exposure at both time points, exhibited no significant changes in ambulatory BP (60)

Concerning other measures of WS, one study followed 3200 men and women for 8 years and found a significantly positive association between change in job demands but not decision latitude and incident hypertension (measured by office BP levels over 160/95 or start of using antihypertensive medication during follow-up) (61). Another study followed 2357 men and women for 20 years and found that worrying about keeping ones job was significantly associated to the development of hypertension (defined as start of using antihypertensive medication during follow-up) among men, but not among women (62). In another study, a low workplace social capital was found to be associated with chronic hypertension, based on health insurance examination, among 11 777 men but not among 49 145 women in a study with 3.5 years of follow-up (55). The same gender difference was found when evaluating low feeling of justice at work and incident hypertension measured by office BP or start on antihypertensive treatment among 4398 men and 1923 women followed for 18 years (18). The opposite gender difference was nevertheless found regarding low job satisfaction in a cohort of 2511 men and 443 women (33). Thus, the dissatisfied women were more in risk of increased office DBP after 4–7 years, whereas no influence of job satisfaction was found on office DBP among the included men (33). Also effort–reward imbalance has been found associated with increased risk of ambulatory BP ≥135/85 among women over 45 years old, while no association was found among younger women or among men of any age (63).

##### Conclusion

Marital stress and perceived stress might have an influence on BP, while an association with distress was not supported. Evaluating job strain, most studies not separating their results by gender did not support an association between job strain and increase in BP, whereas the studies taking gender into account supported job strain as a risk factor for increased BP among men but not among women. Measuring other areas of WS, most studies divided the results by gender and found that worrying about keeping ones job, low workplace social capital, and low feeling of justice seemed associated with increased BP among men, while dissatisfaction with one's job and effort–reward imbalance seemed more associated with increased BP among women. This indicates that different types of WS affect the BP differently between genders. An obstacle when comparing the studies evaluating BP was the differences in methods used for evaluating BP, e.g. office or ambulatory BP or start of using antihypertensive treatment. The ways used for evaluation are highlighted in Table 5.

## Discussion

Based on this literature survey, and with the reported inclusion and exclusion criteria for evaluation, we find that the majority of the studies included support an association between chronic psychosocial stress and the development of MES. With respect to weight gain stress from relationships, generally perceived stress and distress are overall supported as risk factors while the studies on WS showed conflicting results. Too few studies evaluating the development of dyslipidemia were available to draw a conclusion; however, a trend toward a positive association was present. With respect to DM2, the prospective studies supported perceived stress and distress as risk factors among men, but not among women. Regarding WS and DM2, the results were ambiguous; however, there was a tendency of no association. With respect to hypertension, perceived stress but not distress seemed associated with increased BP. Regarding WS and development of hypertension, most studies found some association, mainly among men. Intriguingly the association between the different measures of WS (e.g. job strain and effort–reward imbalance) and development of hypertension differed between men and women.

To our knowledge, this is the first systematic review evaluating chronic stress as a risk factor for MES, which precluded comparisons with previous systematic reviews.

On the individual elements of MES, previous reviews and meta-analyses have been published: a meta-analysis of longitudinal studies on stress as a risk factor for adiposity found overall stress to be associated with increasing adiposity (*r*=0.014, *P*<0.05) (64). However, when dividing the studies into groups of general stress and WS (job strain), neither stress groups alone showed significant associations with increasing adiposity. The meta-analyse focused more on exposure to external stressors rather than on the emotional state of stress and therefore included and excluded differently in relation to this review. Another review pooled data from 13 prospective cohort studies on the association between WS and weight gain (65). In line with the results of the Whitehall II study, they found a bidirectional association between job strain and obesity in the cross-sectional data; however, this was not tested in the longitudinal data (32, 65). In the longitudinal data, the study found that neither job strain at baseline nor continued job strain throughout follow-up was associated with obesity at follow-up, while development of job strain during follow-up was associated with becoming obese at follow-up (65). The results of these analyses have, however, been disputed due to methodological issues (66).

Regarding DM2 in line with this review, a review from the European Depression in Diabetes Research Consortium from 2010 found prospective epidemiological studies to support an association with general emotional stress while conflicting results were found regarding WS (67). Concerning DM2 and WS, a meta-analysis from 2012, including both cross-sectional and prospective observational epidemiological studies, supported the finding of a trend toward no association (68).

On the association between stress and the development of elevated BP, a systematic review of observational studies from 2009 included ten cohort studies and four case–control studies and found a trend for a positive association between chronic stress and elevated BP (69). Regarding job strain, another review found some association between job strain or job strain change and ambulatory BP change when including three longitudinal studies (70). Another recent review on psychosocial work factors and BP has included 40 cross-sectional, 12 prospective studies, and two case–control studies and found that approximately half of the studies supported a significant adverse effect of psychosocial work factors on BP(71). A more consistent adverse effect was observed among men as compared with women (71).

Theoretically, it is plausible that psychological stress induces MES. The pathway is probably multifactorial but could be explained either through stress-induced changes in the HPA axis or/and through stress-induced change in behavior. Considering hormonal changes a stress-induced hyperactivity of the HPA axis may explain the association between stress and all elements of MES: first, excess amounts of glucocorticoids enhance hepatic gluconeogenesis and inhibit the secretion and action of insulin leading to insulin resistance (72, 73). Second, glucocorticoids cause differentiation and proliferation of adipocytes and redistribution of fat, resulting in central adiposity, as it is seen in patients with Cushing's syndrome (74). Third, glucocorticoids decrease lipoprotein–lipase activity which lowers the level of HDL cholesterol and therefore skews the lipid-axes (75). Fourth, elevated levels of glucocorticoids are widely recognized to induce hypertension (76).

A change in behavior, due to chronic stress, may also be the pathway to MES. Kouvonen *et al*. (77) have found a dose dependency between WS (effort–reward imbalance) and the number of health risk behaviors (smoking, BMI >25, physically inactivity, and heavy drinking). People with perceived stress have been shown to spend less time on physical activity and change diet toward eating products richer in fat and sugar (78). Furthermore, stress reduces the probability of success with quitting smoking or reduce alcohol intake (37).

Human characteristics and coping mechanisms differ between individuals, causing them to react differently to stressors. The stress response therefore varies greatly among individuals and both acute and chronic stressors might be modulated with respect to personal factors. The impact of diversity in human characteristics should therefore not be underestimated when interpreting the results of this review. Several studies included finds differences between genders. This gender difference could be explained by women tending to choose other coping mechanisms than men when feeling stressed (79). Also women more often than men generally appraise everyday situations as stressful (79). Considering WS, an explanation for the gender difference could be that men and women have different occupational trajectories and have different effects of social support at work (71).

Overall, this review support that psychological stress is a causal factor for developing MES, while stress as a causal factor for the individual elements of MES shows more ambiguous results. This could indicate that MES as a defined syndrome combines the individual elements to a higher degree than just the sum of the individual elements. This argues for keeping MES as a clinical relevant phenotype even though the risk due to MES is no greater than the sum of the individual risk factors of the syndrome (80).

### Strengths and limitations of the studies included

Most of the studies included had the qualities of following a large cohort over a long time. However, the studies included in this review had a varying quality. Most studies measured the stress exposure at baseline and the MES outcome at the end of follow-up though both the level of stress as well as the MES variables may have fluctuated during this period. Likewise, most studies adjusted for baseline status on physical activity, smoking, and alcohol consumption. However, this may also have changed over time as a response to changes in stress level. Overall, the included studies had a disparity in the adjustments for variables acting as covariates, confounders, or mediators, which could influence the results. Most studies included marital status, socioeconomic, and behavioral factors as physical activity. However, only one study included hours of sleep (38), though stress has been shown to be a strong predictor of sleep duration, and both short and long sleep durations are associated with elevated BMI (81). Likewise, only few studies adjusted for other exposures of stress when measuring WS and vice versa even though adjustment for the stress caused by stressors outside of the area in focus may have given a more adequate picture of the impact of the stress studied. To give an overview of the quality of the studies on this point, the variables included in the adjusted analyses are included in the Tables 1, 2, 3, 4 and 5.

### Strengths and limitations of this review

The strengths of this review are the overview of studies covering the area of chronic stress and both the MES as a syndrome as well as the individual components of the MES. To our knowledge this is the first review to give an overview of both MES as a syndrome and the elements of MES. The prospective design of the studies included provides the best basis for assessing causal relationships. Furthermore, the inclusion of studies measuring both stress in general terms, WS, and distress is strength of this review. We think, however, that it is an advantage that we in the results sections have separated the items to give a more nuanced picture of the area.

A limit specifically to this review is that the majority of studies were excluded since they were not prospective of nature, leaving only a small number of studies available for inclusion especially for dyslipidemia. Thus, one can argue that this form of systematic review is not generalizable, or that the literature is not ready for such a review. However, we believe that the 39 studies included represent sufficiently large populations for conclusions to be valid. Also many details harbored in the studies included are omitted. This was chosen for the purpose of magnitude of this review, and we believe enough details are included to cover the essential information.

The heterogeneity of the included studies concerning both exposure, outcome, control for confounding factors, and gender distribution presents a major limitation and is the reason why this is a narrative rather than a quantitatively systematic review. Also based on the variety in control for confounding factors, we decided to include the ‘must adjusted’ results, which may also represent a limit to the study.

The role of psychosocial stress as a risk factor for MES and the different elements of MES are still unsettled since overall the present evidence for an association is limited. In this context, it is essential to realise that measuring stress at baseline and reporting changes over many years is a fundamental problem, since the burden might fluctuate over time. However, since a consensus on MES being a risk factor for the development of ischemic heart disease, the current evidence level seems to be sufficient to recommend an increased focus on stress-reducing acts both in workplaces and in private life. Prospective studies with specific interventions in order to reduce chronic stress are warranted as is studies focusing on the mechanisms linking chronic psychosocial stress and incident MES. In future studies, a single measure of stress, if ever possible, and a single measure of MES should be preferred.

## Figures and Tables

**Box 1:**
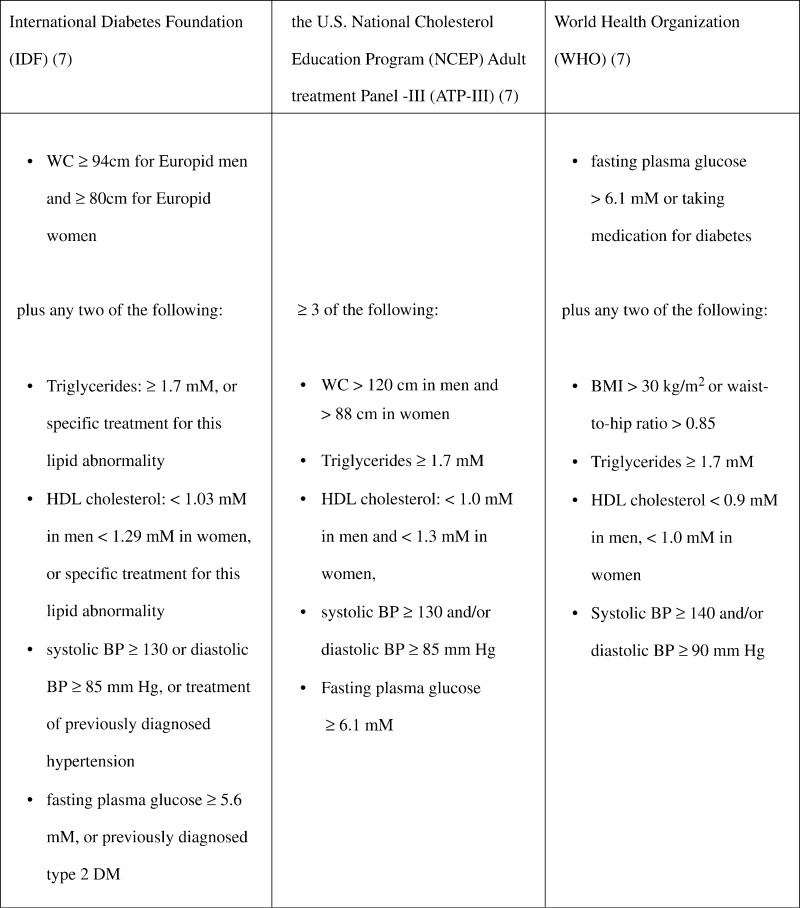
Clinical criteria for the metabolic syndrome.

**Table 1:** Stress and the metabolic syndrome, prospective cohort studies.

**References**	**Study design**	**Sample size**	**Exposure**	**Outcome**	**Adjusted for**	**Groups compared**	**Most adjusted results**
(15)	4 years cohort	216 couples	Six questions on marital adjustment	MES as defined by the NCEP ATP III	Gender, age, income, current and previous smoking, alcohol use, depressive symptoms, physical activity, and baseline MES criterias	Partner reported marital adjustment (higher score, poorer marital adjustment)	Men: OR (95% CI), 1.02 (0.39–2.68)
**Women: OR (95% CI), 10.2 (1.54–68.2)**					
The ELSA study						Actor reported marital adjustment	Men: OR (95% CI), 0.84 (0.21–3.41)
Women: OR (95% CI): 0.37 (0.83–1.67)
(19)	5 years cohort	1408 men and 1558 women	Job Strain Q	MES as defined by the NCEP ATP III	Age, race, physical activity, smoking status, education, family income, daily alcohol consumption, and depression score	High job strain vs low job strain	Men: HR (95% CI), 1.8 (0.9–3.6)
The CARDIA study	**Women: HR (95% CI), 2.2 (1.0–4.6)**
(16)	6.4 years cohort	466 men and women	General health Q	MES as defined by NCEP ATP III	Age, gender, psychological distress, socio-economic status, smoking status, use of alcohol, leisure time physical activity, hsCRP, and General health Q12	High psychological distress vs low psychological distress	**Men and women: OR (95% CI), 1.81 (1.03–3.18)**
A study cohort of middle aged subjects from Finland
(18)	18 years cohort	4398 men and 1923 women	Justice at work scale	MES as defined by NCEP ATP III	Age, ethnicity, and employment grade	Low justice at work vs high justice at work	**Men: HR (95% CI), 0.75 (0.63–0.89)**
The Whitehall II study cohort	Women: HR (95% CI), 0.88 (0.67–1.17)
(7)	15 years cohort	523 women	Perceived stress scale	MES as defined by WHO, NCEP ATP III, and IDF	Age, physical activity, alcohol consumption, smoking status, use of HRT, and level of education	Perceived stress vs no perceived stress	**Women: OR (95% CI), 1.59 (1.11–2.30)**
The Healthy Women Study Cohort							Women: OR (95% CI), 1.19 (0.96–1.47)
Women: OR (95% CI), 1.11 (0.90–1.36)
(17)	14 years cohort	10 308 men and women	Job demand control Q	MES as defined by NCEP ATP III	Age, employment grade, and health behaviours	Greater than or equal to three exposures of job strain vs no exposure of job strain	**Men and women: OR (95% CI), 2.29 (1.27–4.12)**
The Whitehall II study cohort
(14)The Healthy Women Study Cohort	11.5 years cohort	413 women	Study specific seven-item measure of marital quality	MES as defined by NCEP ATP III	Age, baseline metabolic syndrome, length of follow-up, race, education, smoking history, hormone use, physical activity, alcohol consumption, depression, anxiety, and social support	Marital dissatisfied vs marital satisfied	**Women: OR (95% CI), 3.02 (1.46–6.24)**

Bold indicates significant results. OGTT, oral glucose tolerance test; CVD, cardiovascular disease; FPG, fasting plasma glucose; FBG, fasting blood glucose; PG, plasma glucose.

**Table 2:** Stress and adiposity, prospective cohort studies.

**References**	**Study design**	**Sample size**	**Exposure**	**Outcome**	**Adjusted for**	**Groups compared**	**Most adjusted results**
(29)	5–7 years cohort	7332 men and women	Job content Q	Weight gain	Age, baseline weight, alcohol consumption, smoking, physical exercise, limiting longstanding illness, common mental disorders, and employment status at the follow-up	Low job strain vs high job strain	Men: OR (95% CI), 1.07 (0.70–1.63)
The Helsinki Health Study cohort	Women: OR (95% CI), 1.15 (0.95–1.38)
(35)	3 years cohort	136 men and 3647 women	COPSO Q	BMI gain	Analyses on role conflicts were adjusted for: age, cohabitation, type of work position, seniority, and physical work demands. No adjustments were made regarding meaning of work	Meaning of work	Men: OR (95% CI), 1.1 (0.75–1.62)
The COPSOQ-study						Women: OR (95% CI), 1.02 (0.95–1.09)
				BMI loss			**Men: OR (95% CI), 1.95 (1.04–3.66)**
						Women: OR (95% CI), 1.07 (0.97–1.19)
				BMI gain		Role conflicts	Men: OR (95% CI), 1.00 (0.74–1.35)
						**Women: OR (95% CI), 1.13 (1.06–1.19)**
				BMI loss			Men: OR (95% CI), 0.84 (0.54–1.31)
						Women: OR (95% CI), 1.02 (0.93–1.11)
(26)	2 years cohort	3224 men and women	COPSO Q	Change in BMI	Age, sex, education, personal annual income, leadership responsibilities of other employees, shiftwork, number of stressful life events in the last 6 months, neuroticism and extraversion, and loneliness	Higher demand	*B*-estimate (95% CI)
The PRISME study						Lower decision latitude	0.16 (−0.14 to 0.45)
						Lower social support	−0.17 (−0.64 to 0.30)
						Higher effort–reward imbalance	−0.003 (−0.30 to 0.24)
					0.11 (−0.59 to 0.80)
(20)	11.2 years cohort	3703 men and women	Close person Q	>10% increase in BMI	Gender, age, marital status, ethnicity, baseline BMI, employment grade, smoking status, moderate physical activity, vigorous physical activity, daily fruit and vegetable consumption, and common mental disorder	Per one-unit increase in the negative aspects score	**OR (95% CI), 1.06 (1.02–1.10)**
The Whitehall II study cohort		3224 men and women	>10% increase in waist circumference (WC)	Per one-unit increase in the negative aspects score	**OR (95% CI), 1.06 (1.02–1.10)**
(16)	6.4 years cohort	466 men and women	General health Q	WC >102 cm in men or >88 cm in women	Baseline value of psychological distress	High psychological distress vs low psychological distress	**OR (95% CI), 2.5 (1.5–4.0)**
A study cohort of middle aged subjects from Finland							
(27)	2 years cohort	52 men and 20 women	Job demand control Q	Change in BMI	Age, gender, baseline BMI, and education	Job-demand control imbalance	No significant association
A cohort of employees from a Swiss service provider			Effort–reward imbalance Q			Effort–rewards imbalance	No significant association
(25)	4 years cohort	1670 men and women	Kessler 6 scale on psychological distress	Change in BMI	Age, sex, country of birth, marital status, income, alcohol consumption, and smoking	Low psychological distress vs high psychological distress	Decreased BMI: OR (95% CI), 1.08 (0.69–1.70)
The longitudinal study of Australian Children cohort							**Increased BMI: OR (95% CI), 1.38 (1.00–1.90)**
(18)	18 years cohort	4398 men and 1923 women	Justice at work Q	WC >120 cm in men or >88 cm in women	Age, ethnicity, and employment grade	Low justice at work vs high justice at work	**Men: HR (95% CI), 0.68 (0.56–0.82)**
The Whitehall II study cohort							**Women: HR (95% CI), 0.80 (0.64–0.98)**
(22)	13 years cohort	416 men and 757 women	Perceived stress scale	Change in BMI	Stress, age, baseline BMI, smoking, education, occupation, and financial strain	Highest tertile of perceived stress vs lowest tertile of perceived stress	Men: *β* (s.e.m.)=−0.46 (2.37), *P*=0.846
The Pitt County study							**Women: *β* (s.e.m.)=3.49 (1.76), *P*=0.048**
(36)	5 years cohort	4424 men and 5488 women	Job content Q	Change in BMI	Age, smoking, education level, marital status, country of birth, exercise pattern, and baseline BMI quartiles	Longstanding job strain vs no job strain	**Women: 40–59 years old, *P*<0.019**
							Women: 18–39 and 60–80 years old, NS
							Men all ages, NS
(21)	9.2 years cohort	1355 men and women	Study specific scales on job-related demands, perceived constraint in life, strain in relations with family, strain in relation with spouse/partner, and strain in relation with friends	Change in BMI	BMI, age, race, income, generalized anxiety disorder, panic attack, depression, smoking, diabetes, self-rated health, and self-rated relative health	Job-related demands	**Men: *β* (s.e.m.)=0.16 (0.04), *P*<0.001**
The MIDUS study							**Women: *β* (s.e.m.)=0.18 (0.05), *P*<0.001**
						Strain in relations with family	Men: *β* (s.e.m.)=0.04 (0.04), *P*=0.34
							**Women: *β* (s.e.m.)=0.08 (0.03), *P*=0.016**
						Perceived constraint in life	Men: *β* (s.e.m.)=−0.001 (0.02), *P*=0.96
							**Women: *β* (s.e.m.)=0.06 (0.02), *P*<0.001**
(30)	10 years cohort	328 men and women	Study specific question about mental strain at work	Weight gain >15 kg	Age, BMI, occupational status, and education	Increased job stress vs stable or decreased	Men: OR (95% CI), 0.8 (0.4–1.4)
A cohort of employees from the engineering industry in Finland							Women: OR (95% CI), 2.0 (0.9–4.8)
	28 years cohort	305 men and women	Effort–reward imbalance			Low effort–reward imbalance vs high effort reward imbalance	Men: OR (95% CI), 1.7 (0.7–4.4)
							Women: OR (95% CI), 0.6 (0.2–2.6)
(31)	6 years cohort	2200 men and 1371 women	Job demand control Q	Change in BMI above the 75th percentile	Age, sedentary job, shift work, smoking, alcohol, exercise, education, and marital status	High job strain vs low job strain	Men: OR (95% CI), 1.23 (0.95–1.59)
A cohort of employees from a factory in Japan							Women: OR (95% CI), 0.92 (0.66–1.29)
				Change in WC above the 75th percentile			**Men: OR (95% CI), 1.39 (1.07–1.79)**
							**Women: OR (95% CI), 1.78 (1.26–2.52)**
(34)	19 years cohort	6895 men and 3413 women	Job demand control Q	Change in BMI	Age, height, employment grade, education, and health behaviors	Over three episodes of iso-strain vs no iso-strain	**Men: OR (95% CI), 1.92 (1.13–3.24)**
The Whitehall II study cohort							**Women: OR (95% CI), 3.38 (1.16–9.80)**
				Change in WC		Over three episodes of iso-strain vs no iso-strain	**Men: OR (95% CI), 1.46 (1.14–1.87)**
							Women: OR (95% CI), 2.26 (0.78–6.54)
(32)	5 years cohort	5547 men and 2418 women	Modified version of the job demand control Q		Age, employment grade, and baseline BMI	The association between job strain at baseline and BMI at follow-up	Men: *B* (*P* value): 0.005 (0.82)
The Whitehall II study cohort							Women: *B* (*P* value): 0.062 (0.14)
				Weight gain among those with highest quartile of baseline BMI		Per 1 s.d. increase in job strain	**OR (95% CI), 1.22 (1.06–1.41)**
				Weight gain among those with bottom quartile of BMI at baseline		Per 1 s.d. increase in job strain	OR (95% CI), 0.88 (0.76–1.01)
				Weight loss among those with highest quartile of BMI at baseline		Per 1 s.d. increase in job strain	**OR (95% CI), 0.82 (0.71–0.94)**
				Weight loss among those with bottom quartile of BMI at baseline		Per 1 s.d. increase in job strain	OR (95% CI), 1.14 (0.99–1.32)
(28)	25.6 years cohort	545 men and 267 women	Work demands and job control Q	Mean BMI	Sex, age, and baseline BMI	Low vs high job strain	*P*=0.151
A cohort of employees from factories in Finland			Q on effort–reward imbalance			Low vs high effort–reward imbalance	***P*=0.002**
(33)	4–7 years cohort	2511 men and 443 women	Job satisfaction measured by the Reeder Stress Inventory Q	Change in BMI	Age and occupational factors	Association between job satisfaction and change in BMI: dissatisfied with job at both baseline and follow-up	Regressions coefficient (95% CI)
A cohort of employees from workplaces in Scotland							Men: −0.44 (−0.89 to 0.00)
							Women: 1.35 (−0.34 to 3.01)
(24)	15 years cohort	2152 men and 2721 women	Study specific Q on stress of daily activities	Weight gain >10 kg	Age, BMI, education, dieting, alcohol consumption, and smoking pregnancy	High level of stress vs low level of stress	Men, 18–29 years: OR (95% CI), 1.06 (0.56–2.03)
							Women, 18–29 years: OR (95% CI), 1.13 (0.63–2.04)
							**Men, 30–54 years: OR (95% CI), 2.77 (1.06–7.26)**
							Women, 30–54 years: OR (95% CI), 1.35 (0.68–2.66)
(23)A cohort of firefighters, paramedics and fire service administrator	7 years cohort	438 men	Self-reported stress level	Weight gain	No adjustments	Self-reported stress level	Men: correlation between self-reported stress level at baseline and weight change, NS
A cohort of firefighters, paramedics and fire service administrator							

**Table 3:** Stress and dyslipidemia, prospective cohort studies.

**References**	**Study design**	**Sample size**	**Exposure**	**Outcome**	**Adjusted for**	**Groups compared**	**Most adjusted results**
(16)	6.4 years cohort	466 men and women	General health Q	Triglycerides >1.7 mM	Baseline value of psychological distress	High psychological distress vs low psychological distress	OR (95% CI), ∼1.7 (0.9–3.2)
A study cohort of middle aged subjects from Finland				HDL cholesterol <1.03 mM in men and <1.29 mM in women			**OR (95% CI), ∼2.5 (1.4–4.0)**
(18)	18 years cohort	4398 men and 1923 women	Justice at work Q	Triglycerides >1.7 mM or on lipid lowering medication	Age, ethnicity, and employment grade	Low justice at work vs high justice at work	**Men: HR (95% CI), 0.82 (0.73–0.92)**
The Whitehall II study cohort							Women: HR (95% CI), 1.14 (0.2–1.41)
				HDL cholesterol <1.03 mM in men and <1.3 mM in women, or on lipid lowering medication			**Men: HR (95% CI), 0.85 (0.74–0.98)**
							Women: HR (95% CI), 1.04 (0.84–1.30)
(37)	10 years cohort	7066 men and women	Two questions on stress each rated on a four-point likert scale, combined into a seven-point stress score	Change from normal to high cholesterol (total cholesterol </>6.22 mM)	Sex, age, education, and marital status	Low stress vs high stress	Men and women: OR (95% CI), 0.88 (0.68–1.15)
The Copenhagen City Heart Study							
(28)	25.6 years cohort	545 men and 267 women	Work demands and job control Q	Serum total cholesterol	Sex, age, baseline cholesterol	Low vs high job strain	**Men and women, *P*=0.05**
A cohort of employees from factories in Finland			Q on effort–reward imbalance			Low vs high effort–reward imbalance	**Men and women, *P*=0.033**

**Table 4:** Stress and diabetes mellitus type 2 (DM2), prospective cohort studies.

**References**	**Study design**	**Sample size**	**Exposure**	**Outcome**	**Adjusted for**	**Groups compared**	**Most adjusted results**
(44)	8 years (women) – 10 years cohort (men)	2227 men and 3205 women	Demand-decision latitude Q	Incident pre-diabetes or DM measured with OGGT	Age, educational level, psychological distress, family history of diabetes, BMI, physical activity, smoking, and civil status	Job strain vs no job strain	**Men: OR (95% CI), 0.5 (0.3–0.9)**
Stockholm Diabetes Prevention Program						High demands/low decision latitude yes vs no	Women: OR (95% CI), 2.1 (0.9–4.8)
							Men: OR (95% CI), 0.8 (0.4–1.7)
						**Women: OR (95% CI), 4.2 (2.0–8.7) **
(40)	35 years cohort	7251 men	One Q on self-perceived stress during the last 5 years	Incident DM from registries as principal or secondary diagnosis	Age, socio-economic status, BMI, SBP, use of anti-hypertensive medication, and physical inactivity	Permanent stress vs no stress	**Men: HR (95% CI), 1.45 (1.20–1.75)**
Multifactor Primary Prevention Trial Study							
(41)	5 years cohort	3759 men and women	Perceived stress Q	OGTT	Age, education, physical activity, smoking, alcohol consumption, sedentary behavior, adiposity, baseline SBP, triglycerides, HDL cholesterol, and fasting blood glucose	Highest level of perceived stress vs lowest level of perceived stress	Men: OR (95% CI), 1.18 (0.73–1.90)
The Australian Diabetes, Obesity and Lifestyle Study							**Women: OR (95% CI), 1.72 (1.07–2.76)**
(43)	18 years cohort	9514 men and women	General health Q	Self-reported DM	Age, female sex, marital status, education level, annual household income, energy, health status, health problems, activity, and smoking	High psychological distress vs low-psychological distress	Men and women: HR (95% CI), 1.10 (0.91–1.34)
The British Household Panel Survey							
(49)	9 years cohort	3691 men and 3752 women	Job content Q	Incident DM classified as respondent with one hospital admission with a DM diagnosis, or two physician service claims with a DM diagnosis	Age, immigration status, ethnicity, marital status, living location, and education. Baseline heart disease, hypertension, and depression. Activity limitations at work due to health problems, shift schedule, weeks worked, multiple jobs, physical activity at work, BMI, smoking, alcohol, leisure time physical activity, fruit, and vegetable consumption	High job control vs low job control	Men: HR (95% CI), 0.92 (0.56–1.51)
The Canadian Community Health Survey						High psychosocial demands vs low psychosocial demands	**Women: HR (95% CI), 2.04 (1.15–3.61)**
							Men: HR (95% CI), 0.77 (0.48–1.23)
							Women: HR (95% CI), 0.76 (0.43–1.33)
(51)	3.4 years cohort	5843 men and women	Job demands Q	FPG ≥7.0 or HbA1c ≥6.5 or physician diagnosed DM, or use of diabetic medication	Age, gender, education, family history of diabetes, smoking history, sport intensity, and depressive symptoms	Logistics regressions predicting the incidence of diabetes by perceived job control	Men and women: OR (95% CI), 1.05 (0.85–1.15)
Study cohort from Sourasky Medical Center (Tel Aviv)							
(46)	18 years cohort	3689 men and 1449 women	Job strain Q	OGTT ≥11.1 mM or FPG ≥7.0 or reported previously diagnosed DM or use of diabetic medication	Age, employment grade, diet pattern, alcohol consumption, physical activity, smoking status, SBP, triglycerides, and HDL cholesterol	Job strain in non-obese vs no job strain in non-obese	**Men: HR (95% CI), 0.70 (0.53–0.93)**
The Whitehall II study cohort						Job strain in obese vs no job strain in non-obese	Women: HR (95% CI), 1.18 (0.63–2.10)
							Men: HR (95% CI), 1.05 (0.63–1.75)
							**Women: HR (95% CI), 2.01 (1.06–3.82)**
(16)	6.4 years cohort	466 men and women	General health Q	FPG ≥5.6 mM	Baseline value of psychological distress	High psychological distress vs low psychological distress	Men and women: OR (95% CI), 1.2 (0.75–2.0)
A study cohort of middle aged subjects from Finland							
(18)	18 years cohort	4398 men and 1923 women	Justice at work Q	FPG >6.1 mM or on antidiabetic medication	Age, ethnicity, and employment grade	Low justice at work vs high justice at work	Men: HR (95% CI), 1.09 (0.87–1.36)
The Whitehall II study cohort							Women: HR (95% CI), 0.80 (0.54–1.19)
(37)	10 years cohort	7066 men and women	Two questions on stress	Self-reported incident DM	Sex, age, education, and marital status	High level of stress vs low level of stress	**Men: OR (95% CI), 2.36 (1.22–4.59)**
The Copenhagen City Heart Study							Women: OR (95% CI), 0.80 (0.33–1.91)
(38)	10 years cohort	55 826 men and women	Perceived mental stress Q	Self-reported incident DM	Age, BMI, smoking status, alcohol consumption, family history of DM, physical activity, history of hypertension, coffee consumption, type A behavior, and hours of sleep	High level of stress vs low level of stress	**Men: OR (95% CI), 1.36 (1.13–1.63)**
The JPHC Cohort Study							Women: OR (95% CI), 1.22 (0.98–1.51)
(42)	10 years cohort	2127 men and 3100 women	Psychological distress rated on a five question-Q	Incident DM, defined as OGTT ≥11.1 or FPG ≥7.0 or diagnosed DM	Age, BMI, family history of diabetes, smoking, physical activity, and socio-economic position	Highest level of distress vs lowest level of distress	**Men: 2.2 (1.2–4.1)**
The Stockholm Diabetes Prevention Program							Women: 0.5 (0.2–1.2)
(39)	3.2 years cohort	128 men	Perceived stress Q	Incident DM, defined as OGTT ≥11.1 or FPG ≥7.0 or non-fasting plasma glucose level >11.1 mM	Age, BMI, SBT, ALT, LDH, γ-GTP, ALP, protein, creatinine, triglyceride, HDL-C, LDL-C, uric acid, S-amylase, ESR, WBC, hgb, FBG, urinary protein, night duty, blue-collar job, administrative position, business bachelor, stress in daily life, satisfaction with lifestyle, fatigue, alcohol drinking, and current smoking	Stress in daily life vs no stress in daily life	**Men: HR (95% CI), 3.82 (1.09–13.35)**
Japanese company employees cohort							
(48)	5.7 years cohort	62 574 women	Job demand-control Q	Incident DM defined as one or more classic diabetic symptoms and FPG ≥7.8 mM or a random PG ≥11.1 mM or treatment with hypoglycemic medication or OGTT ≥11.1	Age, BMI, family history of diabetes, work hours, rotating night-shift work, hours at work sitting, job support, hours per week of work at home, leisure-time physical activity, smoking, alcohol intake, transunsaturated fat intake, glycemic load, caffeine intake, marital status, number of children, menopausal status, vitamin supplementation, and aspirin use	High demands and low decision latitude vs low demands and low decision latitude	Women: RR (95% CI), 1.11 (0.80–1.52)
Nurses Health Study II from the U.S.							
(50)	10.5 years cohort	8630 men and women	Job demand-control Q	Incident DM defined as OGTT ≥11.1 or FPG ≥7.0 or diagnosed DM	Age, length of follow-up, employment grade, ethnic group, ECG abnormalities, family history of diabetes, BMI, high systolic blood pressure, exercise, smoking, and life events	Effort–reward imbalance	**Men: OR (95% CI), 1.65 (1.0–2.8)**
The Whitehall II study cohort						High job demands vs low job demands	Women: OR (95% CI), 0.93 (0.4–2.0)
							Men: OR (95% CI), 1.07 (0.7–1.6)
							Women: OR (95% CI), 0.52 (0.3–1.1)
(47)	8 years cohort	2597 men	Job demand-control Q	Incident DM defined as OGTT ≥11.1 or FPG ≥7.0 or self-reported diagnosed DM	Age, education, BMI, alcohol consumption, smoking, leisure time physical activity, and family history of NIDDM	Job strain vs no job strain	Men: HR (95% CI), 1.34 (0.50–3.55)
Japanese company employees cohort							

ALP, alkaline phosphatase; ALT, alanine aminotransferase; DM, diabetes mellitus; ESR, erythrocyte sedimentation rate; FPG, fasting plasma glucose; γ-GTP, gamma-glutamyl-transpeptidase; OGTT, oral glucose tolerance test; PG, plasma glucose; SBP, systolic blood pressure; WBC, white blood count.

**Table 5:** Stress and hypertension, prospective cohort studies.

**References**	**Study design**	**Sample size**	**Exposure**	**Outcome**	**Adjusted for**	**Groups compared**	**Most adjusted results**
(63)	3 years cohort	629 men and 966 women	Siegrist Q	Ambulatory systolic blood pressure (SBP) >135 mmHg or diastolic blood pressure (DBP) >85 mmHg	Age, sex, education, income, marital status, BMI, WC, family history of CVD, medication, diabetes, smoking, excessive alcohol consumption, leisure time physical activity, and overcommitment	Never exposed by effort–reward imbalance vs exposed at both baseline and follow-up	Cumulative incidence rate (95% CI):
A cohort of white-collar workers from Quebec City							Men: 1.04 (0.56–1.95)
							Women <45 years old: 1.20 (0.53–2.75)
							**Women >45 years old: 2.78 (1.26–6.10)**
(55)	3.5 years cohort	11 777 men and 49 145 women	The Workplace Social Capital Scale	Chronic hypertension based on health insurance examination, demanding high BP over 6 months followed by 6 months of antihypertensive treatment (office SBP >200 or DBP >95)	Age, sex, SES, marital status, employer, employment time and the size, proportion of male employees, geographic location of the work unit, and co-morbid diabetes or depression	Low vs high self-assessed workplace social capital	**Men: HR (95% CI), 1.38 (1.00–1.90)**
The Finnish Public Sector Study			Job strain Q		Age only	High job strain vs low job strain	Women: HR (95% CI), 1.10 (0.92—1.31)
							Men and women: HR (95% CI), 1.10 (0.97–1.25)
(16)	6.4 years cohort	466 men and women	General health Q	Office BP >130/85 mmHg or use of antihypertensive treatment	Baseline value of psychological distress	High psychological distress vs low psychological distress	Men and women: OR (95% CI), 0.75 (0.4–1.1)
A study cohort of middle aged subjects from Finland							
(18)	18 years cohort	4398 men and 1923 women	Justice at work Q	Office BP >130/85 mmHg or antihypertensive treatment	Age, ethnicity, and employment grade	Low justice at work vs high justice at work	**Men: OR (95% CI), 0.86 (0.78–0.95)**
The Whitehall II study cohort							Women: OR (95% CI), 1.02 (0.87–1.19)
(37)	10 years cohort	7066 men and women	Two questions on stress	Start using antihypertensive medication during follow-up	Sex, age, education, marital status	High stress vs low stress	Men and women: OR (95% CI), 1.39 (1.05–1.84)
The Copenhagen City Heart Study							
(52)	1 year cohort	106 men and 123 women	Job content Q	Change in ambulatory 24 h SBP	Age, gender, ethnic background, premature coronary artery disease, education, BMI, smoking, alcohol use, participation in a stress management or relaxation technique program, regular exercise, and total family income	Job strain	**Men and women: parameter estimate 13.2 (*P*=0.008)**
The Double Exposure Study			Dyadic adjustment scale			Low marital cohesion	Men and women: parameter estimate 0.219 (*P*=0.17)
						Job strain×cohesion	**Men and women: parameter estimate, −0.946 (*P*=0.006)**
(59)	6.5 years cohort	448 men and women	Job demand-control Q	Change in systolic office BP	Age, follow-up time, BP treatment, baseline BP, and years of education	Job strain vs no job strain	**Men: Δ4.6 (2.1) mmHg, *P*=0.045**
The Malmø Diet and Cancer Study				Change in diastolic office BP			Women: Δ2.1 (1.7) mmHg, *P*=0.8
							**Men: Δ4.1 (1.1) mmHg *P*=0.004**
							Women: Δ 0.3 mmHg, (1.0) *P*=0.5
(57)	11 years cohort	5630 men and 2456 women	Job strain: four questions on job demand and 15 on job control	Hypertension defined as ambulatory SBP ≥140 mmHg and DBP ≥90 mmHg or use of antihypertensive treatment	Age, sex, ethnicity, and employment grade	Increase in prevalence (hypertension) per year between low strain, passive, active and high strain groups	Men and women: *P*=0.63
The Whitehall II study cohort						Increase in SBP	*P*=0.63
						Increase in DBP	*P*=0.81
(58)	7.5 years cohort	3483 men and women	Job demand-control Q	Increase in office SBP	Baseline BP	Highest quintile for job strain at baseline and at follow-up vs never exposed	**Men: RR (95% CI), 1.33 (1.01–1.76)**
A cohort of white-collar workers from Quebec City							Women: RR (95% CI), 1.15 (0.93–1.41)
				Increase in office DBP		Highest quintile for job strain at baseline and at follow-up vs never exposed	Men: RR (95% CI), 1.07 (0.84–1.36)
							Women: RR (95% CI), 1.06 (0.85–1.31)
(61)	8 years cohort	3200 men and women	Job demand-control Q	Office SBP >160, DBP >95, or start of using antihypertensive medication during follow-up	Age, BMI, baseline SBP, examination site, education level, and change in BMI	Change in decision latitude	Men and women: OR (95% CI) =1.02 (0.98–1.06)
The CARDIA study						Change in job demands	**Men and women: OR (95% CI) =1.05 (1.01–1.09)**
						Change in ratio of job demands and decision latitude	**Men and women: OR (95% CI) =2.06 (1.01–4.26)**
(56)	5 years cohort	209 men and women	Job demand-control Q	24-h ambulatory SBP	Gender, age, alcohol intake, BMI, occupation, and sodium intake	High job strain at both entry and follow-up vs no job strain at either entry or follow-up	Men and women: SBP, 118±2 vs 120±1 mmHg, NS
A cohort of employees from a chemical company in France				24-h ambulatory DBP			Men and women: DBP, 76±2 vs 77±1 mmHg, NS
(33)	4–7 years cohort	2511 men and 443 women	Job satisfaction measured by the Reeder Stress Inventory Q	Change in office DBP	Age and occupational factors	Association between job satisfaction and change in DBP. Dissatisfied with job at both baseline and follow-up	Regressions coefficient (95% CI):
A cohort of employees from workplaces in Scotland							Men: −1.29 (−3.09 to 0.52)
							**Women: 6.05 (0.05 to 12.05)**
(62)	20 years cohort	2357 men and women	Qs on work-related stressors	Start of using antihypertensive medication during follow-up	Age, BMI, physical activity, alcohol consumption, depression, education, smoking, unemployment, race, and job status	Worried about keeping job vs not worried about keeping job	**Men: OR: 1.6 (1.1–2.2)**
The Alameda county study							Women: OR: 1.0 (0.7–1.5)
(54)	3 years cohort	103 men and women	Dyadic adjustment scale	Increase in 24-h ambulatory BP	Sex, age, BMI, smoking status, alcohol use, practice of relaxation techniques, exercise, and previous antihypertensive medication	Lowest quartile of marital cohesion and increase in ambulatory blood pressure	**Men and women: *sr*^2^=0.045, *P*=0.01**
Cohabiting males or females						Lowest quartile of marital satisfaction and increase in ambulatory blood pressure	Men and women: *sr* ^2^=0.024, *P*=0.05
(60)	3 years cohort	195 men	Job content Q	Change in ambulatory SBP:	Age, BMI, ethnicity, alcohol use, type A behavior, education, and smoking	*F*-test for no significant association between job-strain groups (no–no, no–yes, yes–no, and yes–yes) and mean change in ambulatory BP on different locations	Men: significance of *F*-test:
The work site blood pressure study				At work			*P*=0.14
				At home			*P*=0.26
				While sleeping			*P*=0.96
				Change in ambulatory DBP:			
				At work			*P*=0.07
				At home			*P*=0.10
				While sleeping			*P*=0.17

CVD, cardiovascular disease; SES, socio-economic status.
